# Mismatch of Glucose Allocation between Different Life Functions in the Transition Period of Dairy Cows

**DOI:** 10.3390/ani10061028

**Published:** 2020-06-13

**Authors:** Jonas Habel, Albert Sundrum

**Affiliations:** Department of Animal Nutrition and Animal Health, Faculty of Organic Agricultural Sciences, University of Kassel, Nordbahnhofstr. 1a, 37213 Witzenhausen, Germany; sundrum@uni-kassel.de

**Keywords:** glucose metabolism, immune dysfunction, production diseases, lactational physiology, trade-offs

## Abstract

**Simple Summary:**

The reasons for the development of diseases in the transition period of dairy cows are manifold and highly farm- and cow-specific. Nevertheless, links exist between the degree of negative energy balance (NEB) and disease susceptibility, which suggest a mutual adverse relationship between immune and productive functions. Glucose is the most essential fuel and precursor for both immune cells and mammary epithelial cells (MEC). While the delivery of glucose by the intermediary metabolism is not always able to keep up with whole-body demands, trade-offs between mammary and immune cells emerge. The prioritization of mammary supply during early lactation is a physiologic principle in all mammals. In contrast, tremendous increases in milk yield and the specific demand for glucose in high-yielding dairy cows resulting from decades of selection for milk production override the evolutionary principles of nutrient partitioning. Therefore, high-producing dairy cows face an increased risk of glucose shortages in their immune cells, particularly during early lactation.

**Abstract:**

Immune cell functions such as phagocytosis and synthesis of immunometabolites, as well as immune cell survival, proliferation and differentiation, largely depend on an adequate availability of glucose by immune cells. During inflammation, the glucose demands of the immune system may increase to amounts similar to those required for high milk yields. Similar metabolic pathways are involved in the adaptation to both lactation and inflammation, including changes in the somatotropic axis and glucocorticoid response, as well as adipokine and cytokine release. They affect (i) cell growth, proliferation and activation, which determines the metabolic activity and thus the glucose demand of the respective cells; (ii) the overall availability of glucose through intake, mobilization and gluconeogenesis; and (iii) glucose uptake and utilization by different tissues. Metabolic adaptation to inflammation and milk synthesis is interconnected. An increased demand of one life function has an impact on the supply and utilization of glucose by competing life functions, including glucose receptor expression, blood flow and oxidation characteristics. In cows with high genetic merits for milk production, changes in the somatotropic axis affecting carbohydrate and lipid metabolism as well as immune functions are profound. The ability to cut down milk synthesis during periods when whole-body demand exceeds the supply is limited. Excessive mobilization and allocation of glucose to the mammary gland are likely to contribute considerably to peripartal immune dysfunction.

## 1. Introduction

Animal welfare concerns, as well as the economic implications of the high prevalence of production diseases [1,2,3] and increasing rates of involuntary culling in dairy farming [4], emphasize the need to address possible conflicts of aims between these impacts and the level of milk production. Many scientific investigations are based on the assumption that individual genomic and/or metabolomic differences are able to explain why some cows are both high producing and healthy while others fail to cope [5,6,7,8]. These approaches try to identify cows that are more efficient in digestion, absorption, synthesis and mammary utilization of nutrients. However, they disregard the limitations in the capacity to deal with deficiencies in a highly heterogeneous and dynamically changing environment that is elusive to deterministic approaches. They disregard that immune functionality is fundamental to ensure health, longevity and productivity of dairy cows, as it is not only essential for pathogen elimination but is part of the coordinated reaction of the organism to all kinds of stressors.

After parturition, high-producing dairy cows generally enter a negative energy balance (NEB), because their level of dry matter intake (DMI) does not meet the demands imposed by the onset of milk production [9]. Consequently, they mobilize body tissue to overcome this shortage. Excessive mobilization can lead to a hypercatabolic response described as metabolic stress, associated with the occurrence of subclinical and clinical diseases [10]. The overall energy budget of an organism includes various energy sources, metabolic pathways and interactions between subsystems of nutrient trafficking that make it difficult to evaluate the consequences of sustained overall NEB on metabolic disorders and health. In fact, plasma concentrations of single metabolites vary substantially between individual cows with similar status of NEB in early lactation [11,12,13,14]. Thus, it has been emphasized that we must move “from joules to moles of molecules or groups of molecules” to advance animal nutrition concepts [15].

In dairy cows, the amount of glucose required to fuel milk production outreaches by far energy expenditures of other life functions like reproduction or maintenance [16]. Besides being a precursor for the synthesis of lactose, which is the osmotic regulator of milk volume [17,18], glucose-derived carbon is also found in milk fat and protein [19,20]. Moreover, reduction of nicotinamide adenine dinucleotide phosphate (NADP^+^) through pentose phosphate pathway as well as the production of ATP, which are required for the synthetic processes depend on the availability of glucose. During peak lactation, mammary epithelial cells (MEC) are able to retrieve up to 2.7 kg of glucose per day from the plasma pool at a milk level of 40 kg [16]. On the other hand, cells of the innate and adaptive immune system rely largely on the uptake of glucose and the storage of glycogen, because glucose supports proliferation, survival and differentiation as well as essential functions like phagocytosis and reactive oxygen species (ROS) production [21]. Recent data about dairy cows exposed to lipopolysaccharide (LPS) challenges showed that a fully activated immune system needs 2.5 to 3.1 kg of glucose per day [22]. In other words, immunoactivities in dairy cows can amount to degrees of glucose demands similar to those required for high milk yields. In contrast to monogastric species, ruminants cover their glucose demand almost exclusively through hepatic gluconeogenesis, which seems limited to about 3 kg of glucose at a milk yield level of ~40 kg/day [23]. These considerations and the quantities at hand clearly indicate a competitive situation between milk production and immune defense and give rise to the question of how allocation of nutrients between different tissues and life functions is regulated when essential nutrients become scarce.

## 2. Resource Allocation between Maintenance and Productive Life Functions in Early Lactating High-Producing Dairy Cows

### 2.1. Resource Allocation Theory

According to the resource allocation theory [24], resources including energy and essential nutrients have to be partitioned between all life functions. Common differentiations of life functions that an animal has to fuel include productive processes such as growth, gestation and lactation as well as maintenance functions. However, definitions of maintenance and the requirements of regulatory systems are essentially conceptual, of a qualitative nature and are also imprecise and contradictory throughout the scientific literature [25,26]. However, activities like thermoregulation and immune function are known to impose high demands [27].

As for the relationship between productive and other life functions in dairy cows it was hypothesized that cows with high genetic merit for milk production cover their demand by an increase in feed intake, while cows with low genetic merit—if they do consume more feed—accumulate body reserves [28]. Consequently, maintenance requirements would be unaffected by milk yield, and differences in milk yield could be due to differences in the efficiency of energy and nutrient utilization—a phenomenon that was described as “dilution of maintenance” [28]. Although correlations between yield and intake ranging from 0.46 to 0.65 [29] indicate the strong relationship between these variables, they reveal as well that the increase in feed intake does not keep pace with the increased demand imposed by an increased number of lactocytes in the mammary gland. Accordingly, increases in body weight associated with selection for milk production have been shown to increase maintenance requirements as well [30]. While energy expenditures by visceral organs represent 0.4-fold of the maintenance requirements for nonproductive adults, they increase to 1.2-fold for lactating ruminants [31]. Moreover, increased energy and nutrient demands for milk synthesis may also lead to time constraints since eating and rumination time has to be traded off against all other activities. In fact, there is little sign that high genetic merit cows digest feed more efficiently than low genetic merit cows. It has been shown decades ago that digestive efficiency decreases as feed intake increases [32]. Accordingly, the nonlinear character of the relationship between milk yield and body weight suggest an optimum body weight beyond which the feed-efficiency decreases in specialized breeds [33]. Loncke et al. (2020) recently showed a similar pattern for the efficiency of hepatic glucose synthesis (conversion rate of precursors to glucose), which decreases at high levels of precursor supply [34]. As a result, high-producing dairy cows show increased rates of energy mobilization from body tissues to bridge the gap between the supply of energy from feedstuffs and the energy needed to support milk production along with all other energy-demanding life functions [35].

### 2.2. Homeorhetic and Allostatic Control of Nutrient Partitioning

Because most organs and tissues lack autonomy to control their nutrient access, the organism needs prioritization rules that coordinate nutrient partitioning between different life functions in all situations where demand exceeds supply. In this regard, different concepts of regulation have been established and adapted to dairy cow physiology. In the concept of homeorhesis, nutrient partitioning is described as a function of biological needs that alternates cyclically between storing and mobilization of energy from body tissues and the associated prioritization of reproductive and productive functions [9]. Following this approach, it is a fundamental biological principle that after parturition, mammalian organisms prioritize mammary tissues to provide an adequate supply to the neonate. Dairy production is based on, and takes advantage of, this principle through a performance-oriented selection of animals.

However, environmental and nutritional stressors associated with varying quality and quantity of feedstuffs, social stress, climate variability and extremes, poor hygiene, technical failure, etc. also affect supply and demand of nutrients independent of the physiological state [27,36]. In this regard, it has been emphasized that systematically reviewing the literature from epidemiological studies is unlikely to support understanding of the effects of metabolic imbalances of each cow in her specific genetic and environmental circumstance due to the fact that intricate biological correlations—besides within- and between-herd confounding effects—exist [37].

The concept of allostasis goes beyond the concepts of homeostasis and homeorhesis by assuming dynamic set points emerging from the integration of both the requirements of different tissues at different physiological states and current nutritional, social and housing conditions [38]. In dairy cows, various genotype x environment interactions have been described, including a reduced ability to adapt to unfavorable conditions (plasticity) in cows with high genetic merit for milk production [39]. This suggests that the process of adaptation to such conditions imposes a certain demand for energy itself, which has been described qualitatively as the “allostatic load” of the animal [40]. If the allostatic load becomes too big (allostatic overload), the capacity to cope with additional stressors is reduced and the animal enters a pre-pathological state [41]. However, by considering maintenance functions as costs, they have to be traded off against productivity goals. Following this approach, resource availability for immune cells provided through a balanced resource allocation is a prerequisite for an organism to be able to cope with internal and external stressors.

## 3. Glucose Metabolism to Fuel Milk Synthesis and Immune Functions in Dairy Cows

**Figure 1:**
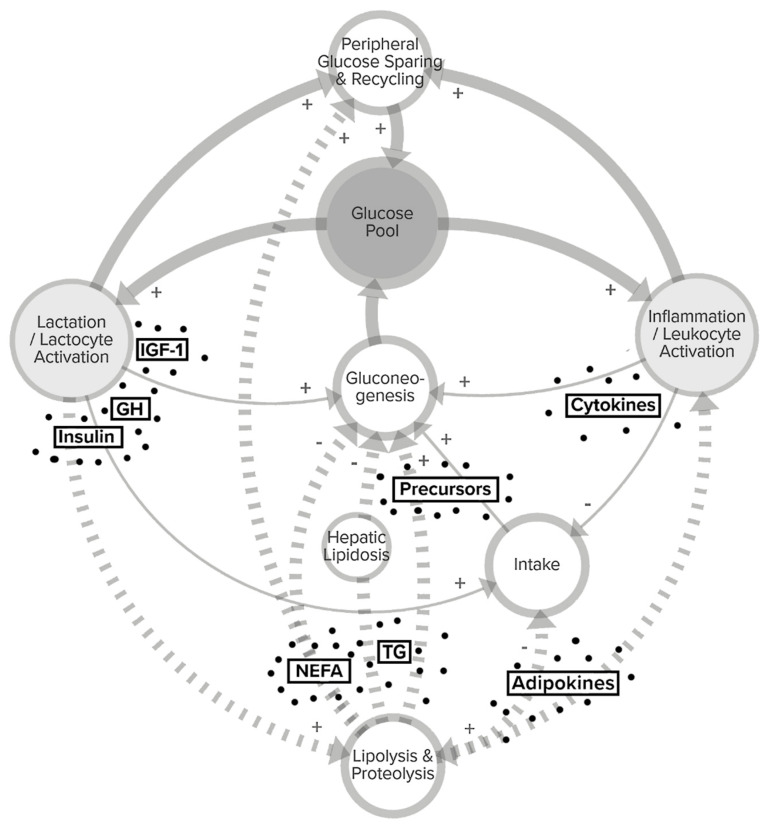
Schematic representation of metabolic pathways related to the glucose balance of dairy cows during lactation and inflammation. Milk synthesis and immune defense rely on an adequate supply with glucose, as it is an important energy source and precursor for lactocytes and leukocytes. To increase overall glucose availability to the respective cell type, inflammatory signals (cytokines) as well as peripartal fluctuation of hormones associated with the somatotropic axis such as growth hormone (GH), insulin and insulin-like growth factor 1 (IGF-1) enhance (+) the rate of gluconeogenesis and affect the level of intake (solid lines, thin), and increase the mobilization of body reserves (dashed arrows). Lipolysis and proteolysis provide endogenous glucose precursors such as alanin and glycerol as well as alternative energy sources like non-esterified fatty acids (NEFA) that help spare glucose in peripheral tissues, where insulin sensitivity is reduced. Because glucose uptake is non-dependent on insulin in both leukocytes and lactocytes, trade-offs for glucose allocation (solid arrows, bold) may arise in situations where inflammation and lactation impose high demands. Limitations may also arise from negative effects (-) of adipokines and cytokines on the hypothalamic regulation of intake and from hepatic accumulation of triglycerides (TG) and NEFA when lipolysis is excessive.

Proinflammatory signaling promotes similar metabolic adaptations, as does the lactational “reprogramming” with the aim of maximizing glucose availability to the respective cell types (Figure 1). These changes are mediated by a network of hormones and affect both mobilization and allocation, including specific regulation of blood flow and receptor expression patterns in peripheral, mammary and immune tissues. In the following chapters, the metabolic processes associated with the onset of lactation (Section 3.1) and those occurring during inflammation (Section 3.2) are discussed separately. Subsequently, trade-offs for glucose between MEC and immune cells in dairy cows are evaluated in Section 4.

### 3.1. Adaptation to Lactation

Morphological changes required for mammary growth, morphogenesis, and milk synthesis are created during gestation, with ductal elongation and lobulo-alveolar development being mediated through high plasma concentration of prolactin, growth hormone (GH) and gonadotropic steroids progesterone and estrogen before parturition [42,43]. Subsequently, high concentrations of GH are known to stimulate galactopoiesis, while prolactin and Insulin-like growth factor 1 (IGF-1) are involved in establishing and maintaining milk synthesis through their effect on epithelial cell differentiation and survival [44,45].

To meet the sudden increase in demand during early lactation, further alterations in the endocrine setup support dairy cows to metabolically adapt to lactation. Besides the above-mentioned hormones, insulin, thyroid hormones, glucocorticoids and the gonadotropic axis are main effectors of the new catabolic physiology and plasma concentrations of these hormones typically fluctuate at the transition from a pregnant to a lactating physiology [46]. Especially changes in the somatotropic axis; i.e., decreased pancreatic secretion of insulin and reduced GH-receptor (GHR) expression in the liver [47] are thought to be major levers of the new catabolic physiology [48,49,50]. Subsequently, hepatic GH resistance and hypoinsulinemia mitigate stimulating effects on hepatic IGF-1 production [51]—a condition that has been described as the “uncoupling of the somatotropic axis” [48].

Insulin signaling is particularly essential for a successful adaptation to lactation by affecting the rate of lipolysis, the rate of uptake and transport of glucose and fatty acids to different tissues, and the expression of key enzymes at the metabolic crossroads of glucose and fatty acid metabolism [52,53]. A main effect of peripartal hypoinsulinemia is the reduction in insulin’s antilipolytic properties, which facilitates the mobilization of nutrients from body reserves by increasing the rates of lipolysis and proteolysis [54]. These processes are accompanied by increased rates of gluconeogenesis, reflected by an increased mRNA amount of the important rate-limiting enzymes pyruvate carboxylase (PC) and phosphoenolpyruvatecarboxykinase (PEPCK) postpartum [55]. Precursors for gluconeogenesis include rumen-derived volatile fatty acids, mainly propionate and, to a lesser extent, circulating C3-bodies like glycerol, alanine and lactate from intermediary metabolism [23]. Increased levels of circulating non-esterified fatty acids (NEFA) resulting from adipose tissue remodeling are taken up proportionally to their plasma level by the liver. Together with a simultaneous lack of oxaloacetate, which is highly used for gluconeogenesis, increased NEFA lead to an accumulation of acetyl-CoA in the liver. Subsequently, hepatocytes are forced to switch acetyl-CoA utilization from complete (Krebs cycle) towards incomplete oxidation (ketogenesis) and/or to re-esterification with subsequent storage of triglyceride in the liver [56]. Besides negative effects of hepatic TG accumulation on general hepatic function, increased levels of β-hydroxybutyrate (BHB) were shown to impair gluconeogenic capacity [57,58]. In cultured bovine hepatocytes, increasing levels of NEFA gradually decrease mRNA levels and catalytic activity of PC and PEPCK [59]. Thus, glucose balance is challenged severely when lipolysis becomes excessive. Although the usefulness of plasma glucose as an indicator of a cow’s metabolic status is particularly contentious due to the tight regulation of glucose homeostasis [60], hypoglycemia is associated with the onset of ketosis, higher first test-day milk production and milk production at 100 days in milk [61].

Moreover, allocation patterns that regulate the flow of nutrients between different tissues within the organism change according to the new dominant physiological state of lactation. Again, these changes are related to the ‘uncoupled’ somatotropic axis and in particular, to the phenomenon of reduced insulin sensitivity in peripheral tissues of postpartum dairy cows [62,63,64,65]. Because glucose receptors prevailing in the mammary gland are mostly non-dependent on insulin while muscle and adipose cells are highly insulin-responsive cell types [66], reduced peripheral insulin sensitivity favors the glucose supply of lactocytes. Simultaneously, mRNA encoding insulin-independent glucose transporter (GLUT) with the highest affinity to glucose (GLUT1) increases strongly in mammary tissues at the onset of milk synthesis [67]. As lactation advances, the mammary gland becomes more insulin-sensitive and insulin-dependent while glucose uptake via GLUT4 increases [68]. In contrast, GLUT1 decreases about 6-fold in mRNA and protein levels in adipose tissue of early lactating cows compared with dried off or late lactating cows [67]. However, the extraction of great amounts of glucose from circulation is promoted primarily through a greater blood flow to the mammary gland, which was found to be stimulated by the characteristic endocrine regulation of lactation [69,70]. In particular, thyroxine is thought to enhance mammary nutrient extraction by increased heart rate and subsequent increases in blood flow [71]. This was identified decades ago as a main determinant of quantitative udder metabolism [16,72]. In more recent studies, blood sampling techniques comparing glucose concentrations from the jugular and mammary vein showed a lower jugular/mammary quotient for glucose concentration in dry and low-yielding cows, while revealing significantly higher levels in high yielding cows [73].

In peripheral tissues, modest reductions [74,75] or no significant changes [67] in the expression of insulin-dependent GLUT 4 in peripheral tissues have been reported in early lactating dairy cows. However, these tissues are aligned to save glucose during that period by shifting their glucose metabolism from complete oxidation towards lactate production. Accordingly, irreversible losses of glucose excluding the loss in milk lactose decreases significantly in the first days after parturition [76]. Together with alanine and glycerol derived from muscle resp. adipose tissue, lactate can recirculate to the liver, where it is supposed to have a higher proportional contribution to gluconeogenesis during early lactation [23].

In summary, a complex endocrine network develops to increase glucose availability to the mammary gland. If precursor supply or hepatic synthetic capacity are inadequate, the sudden increase in mammary demand for glucose at the onset of lactation is the main driver of the hypermetabolic reaction that affects a variety of metabolic pathways, tissues and organs within the organism.

### 3.2. Adaptation to Inflammation

Immune cell activity and inflammation are not only essential for pathogen elimination but are part of the coordinated reaction of the organism to all kinds of stressors, including infective and non-infective, metabolic and environmental stressors. After parturition, dairy cows experience an inflammatory-like status, which is systemically linked to the inherent stress of parturition, social and nutritional changes and the endotoxin-releasing processes of ruminal adaptation and uterine tissue reorganization [77,78]. The response is characterized by a marked increase in plasma concentration of positive acute phase proteins [79]. Their plasma level has been associated with the occurrence of retained placenta, other diseases and impaired reproductive and productive performance during early lactation [80,81]. However, the necessity of some degree of “physiological inflammation” is illustrated by the action of anti-inflammatory drugs that inhibit the synthesis of prostaglandins required to expel the placenta [82]. Following administration of an anti-inflammatory drug after calving, dairy cows have an increased risk of retained placenta (2.5-fold) and metritis (1.5-fold) [83].

**Figure 2:**
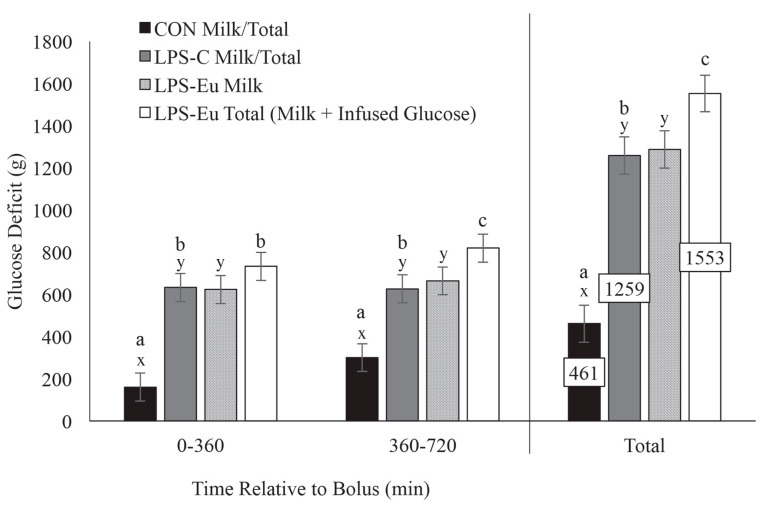
Milk or total glucose deficit from zero to 360, 360 to 720, and accumulated over 720 min in cows administered a bolus of saline (CON), lipopolysaccharide (LPS-C), or lipopolysaccharide accompanied with a euglycemic clamp (LPS-Eu). Different letters (x,y) represent differences between milk glucose deficits (*p* ≤ 0.05). Different letters (a–c) represent differences between total glucose deficits (*p* ≤ 0.05; total glucose deficit = milk glucose deficit in CON and LPS-C cows; total deficit = milk glucose deficit + infused glucose in LPS-Eu cows). Results are expressed as least square means ± standard error of means. Reprinted from Kvidera et al. (2017), Copyright (2017) with permission from Elsevier.

Whatever the origin of inflammation, the accumulation of proinflammatory processes implies a supply with energy that adequately meets the requirements of immune response. Qualitatively, the costs of immune activation include (1) a general elevation of metabolic rates due to a rise in body temperature, (2) reduced nutrient availability following anorexic effects of proinflammatory signaling, (3) the precursors and energy needed to fuel the synthesis of acute-phase proteins and immunoglobulins, (4) altered priorities for nutrient utilization in other tissues, (5) the costs associated with the repair of damaged tissues and (6) increased turnover rates of the leukocyte pool [27]. Although an almost infinite number of possible combinations between metabolic and environmental stressors make it impossible to estimate the current degree and duration of inflammation and immunoactivation and to determine the energy demand of immune cells, some quantification has been performed. For instance, it has been shown that the demand for oxygen, glucose and glutamine increases two- to three-fold during lymphocyte activation [84]. By examining the effect of an infection with nematode larvae on the energy requirement of merino sheep, it was estimated that infection increased the requirement for metabolizable energy by 28% [85]. Even more impressively, Kvidera and colleagues combined an intravenous LPS challenge, a euglycemic clamp and measurement of milk yield reduction in cows of parity 2 or 3 that were at 69 ± 7 days in milk to calculate the demand of a fully activated immune system. The authors estimated that dairy cows may require up to 3.1 kg of glucose per day to mount an acute inflammatory response (Figure 2) [22].

In fact, cells of the innate and adaptive immunity rely largely on the uptake of glucose and the storage of glycogen, because glucose supports proliferation, survival and differentiation as well as essential functions like phagocytosis and ROS production [21]. Moreover, an activation of apoptotic pathways in response to limited glucose uptake in cultured hematopoietic cells was reported [86]. In dairy cows, reduced glycogen concentrations in circulating neutrophils at calving indicate a depletion of glucose depots during this challenging period and are associated with the occurrence of subclinical endometritis and metritis [87]. Although immune cells are able to use alternative energy sources like glutamine and ketone bodies to some extent [88,89,90], the importance of glucose as their main fuel was corroborated by Noleto et al., who found that supplying increasing amounts of glutamine in the absence of glucose was not sufficient to raise the inflammatory response to LPS in endometrial monocytes and macrophages of dairy cows, whereas supplying more glucose was able to increase inflammation in the absence of glutamine [91].

Not surprisingly, leukocytes trigger a number of metabolic pathways that increase the glucose supply to these cells while reducing consumption of glucose by other tissues. First references describing the link between inflammation and insulins actions date far back [92]. By now it is clear that the interplay between proinflammatory and insulin signaling is common to all the mammals [93]. In dairy cows, the effect of continuous and increasing LPS-infusion on whole-body insulin-resistance has recently been demonstrated [94]. T-cells were shown to shift glucose transporter expression from insulin-dependent GLUT4 towards GLUT1 and GLUT3, which are non-dependent on insulin, to maintain glucose disposal during activation [95,96,97]. Inflammatory pathways also promote the transcription of gluconeogenic genes via toll-like receptor 4 (TLR-4) [98]. Macrophages and neutrophils undergo a metabolic switch from oxidative phosphorylation towards glycolysis during activation, thereby increasing their demand for glucose as well as their lactate production [99]. Metabolic reactions to the alterations induced by proinflammatory cytokines further encompass increased rates of lipolysis and proteolysis, that could provide energy for leukocyte functions as well as substrates for gluconeogenesis [100,101]. However, the inflammation-mediated metabolic reprogramming appears very similar to the reprogramming mediated by lactation, both aiming at a maximum supply of glucose for the respective cell functions. On a systemic level, this includes increased rates of gluconeogenesis and reduced glucose consumption in peripheral tissues.

## 4. Trade-Offs for Glucose between Lactocytes and Leukocytes

All mammals favor the supply of nutrients to the mammary gland during early lactation. In contrast, the increases in milk yield and the specific demand for glucose required for high milk yields override evolutionary principles of nutrient partitioning [102]. Because nutritional supply is limited through various factors, e.g., percentage of concentrate in the diet, time to eat, turnover rates in the rumen as well as the synthetic capacity of the liver during this period, most high-producing dairy cows experience a period of glucose shortage. In such situations, trade-offs for glucose between MEC and immune cells are unavoidable as they both rely on this essential substrate. Therefore, a special focus on the allocation dynamics of glucose is necessary during periods when both lactation and inflammation impose high demands.

### 4.1. Peripartal Immune Dysfunction

The phenomenon of reduced immune cell competence is well established in peripartal dairy cows. It is broadly characterized by a dysfunction of PMN, macrophages and lymphocytes, including an impairment of viability, survival, phagocytosis and respiratory burst capacity [103]. Studying gene expression profiles in the bovine mammary gland during stage I and II of lactogenesis, it was found that most of the genes associated with immune response were downregulated at the end of gestation [104]. This is in line with the interpretation of Goff and Horst, who suggested that neutrophil phagocytosis and lymphocyte proliferation begin to be impaired around three weeks before parturition [105]. Moreover, significant changes in lymphocyte subsets occur. Overall number and proliferation of circulating lymphocytes are reduced, while mammary cell number and proliferation peaks around calving [43,106,107,108]. Accordingly, altered immune functions during the dry period are associated with the development of metabolic disease during early lactation [106]. Around parturition, elevated levels of glucocorticoids and decreased plasma levels of oestrogens and progesterone also affect immune response through altered MHC-expression, cytokine production, diapedesis capacity and viability of immune cells [109,110,111]. In summary, the mammary gland prepares for lactation not only by improving functionality but also by suppression of competitive functions, allowing more resources to be used for milk synthesis [104].

### 4.2. Metabolic Stress and the Immune System

The aforementioned relationships suggest that the substantial but transient suppression of immune functions before parturition is related to the physiological adaptation to lactation. However, not only cell number and proliferation, but also functionality of immune cells is impaired strongest when MEC start the abundant synthetic activity of lactogenesis as was demonstrated by the transient loss of expression of vascular factors and antimicrobial chemokines [104,112,113,114]. Accordingly, mastectomized cows had a shorter and less marked immune suppression, including less impairment of oxidative burst capacity and faster recovery of myeloperoxidase activity in neutrophils at calving compared to non-mastectomized cows [115,116]. This indicates that some immunosuppressive effects may be independent from the endocrine changes associated with parturition but related directly to the capacity to synthesize milk. In fact, various effects of severe NEB on immunosuppression have been published and many of them are related to the effects of adipose-tissue remodeling on key molecules involved in glucose and lipid metabolism [117]. For instance, high plasma levels of BHB are negatively correlated with DNA replication and repair in leukocytes [118]. Plasma concentration of NEFA correlate with increased hepatic expression of mRNA encoding proinflammatory cytokines and acute-phase proteins [119]. Increased hepatic uptake of NEFA may also result in increased production of reactive oxygen species (ROS) that carry out important tasks of immune defense by facilitating the destruction of pathogens and enhancing the proinflammatory cascade at physiological plasma concentrations [120]. They can affect the integrity of immune cells, which are very susceptible to peroxidation due to high concentrations of polyunsaturated fatty acids in their membranes. Additionally, ROS generated during inflammation have been proposed to play a role in mediating insulin resistance [121]. If NEFA mobilization and ROS production is excessive, host tissues may fail to mitigate the negative effects of ROS by activation of antioxidant pathways, resulting in severe tissue damage [122]. Leukocyte function is also affected by the shift in fatty acid profile resulting from lipomobilization [123]. Altered concentrations of adipokines postpartum mitigate stimulating effects on chemotaxis and phagocytosis of neutrophils, proliferation of native T-cells and the secretion of cytokines as well as anti-inflammatory effects [124,125], presumably via activation of TLR-4 and nuclear factor kappa-B (NFkB) [74,126].

Accordingly, cows with severe NEB have a reduced ability to clear uterine infection postpartum. The active uterine inflammatory response in these cows was associated with impaired local insulin-receptor signaling [127]. In the mammary gland of lactating dairy cows subjected to a dietary-induced NEB, expression of genes related to proinflammatory signaling via NFkB (AKT1, IRAK1, MAPK9 and TRAF6), IL-8 (e.g., CXCR1/R2) and chemokine signaling (e.g., SOCS2) were downregulated [128].

Nevertheless, experimentally induced negative energy balance in advanced lactation was repeatedly shown to be unable to cause alterations of inflammation and immune cell function that are as severe as those occurring during early lactation [129,130,131]. With regard to the importance of glucose for immune cells, a possible mechanism associated with different reactions to similar NEB could be an increased glucose availability during late lactation, as it was demonstrated that late-lactation induced NEB evoked less severe proportional decreases in plasma glucose concentrations, compared to early-lactation NEB [132,133].

### 4.3. Competition for Glucose between MEC and Immune Cells

As addressed previously, the usefulness of plasma glucose as an indicator of a cow’s metabolic status is unsure due to the tight regulation of glucose homeostasis [60]. However, Graber and colleagues differentiated metabolically robust or vulnerable cows based on the occurrence of various metabolic and (re)productive disorders in previous lactations and identified plasma glucose as the only variable explaining the differences between those groups at both time points investigated (3 weeks before and 4 weeks after parturition) [12]. In another study, plasma concentrations of glucose and insulin during lactation were found to be the single most important predictors related to the development of disease, explaining 36% of the between-cow variability in energy-corrected milk [14].

**Figure 3:**
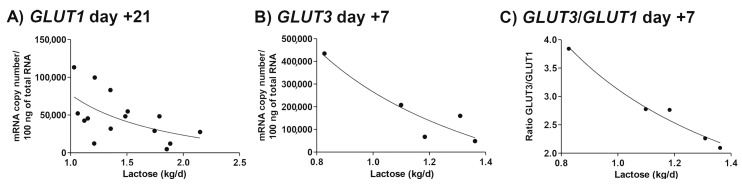
Peripartal monocyte glucose transporter expression is correlated with lactose production. glucose transporter 1 (GLUT1) (**A**) and GLUT3 (**B**) mRNA expression as well as the GLUT3/GLUT1 ratio (**C**) at d + 7 and d + 21 relative to parturition were correlated with milk production data of wk 1 (*n* = 5) and wk 3 (*n* = 15) of lactation, respectively, using Pearson (GLUT1 and GLUT3/GLUT1) or Spearman (GLUT3) correlation. Significant correlations with lactose production (at least *p* < 0.05, r > −0.50) were followed by nonlinear regression analysis (inverse model: Y = B0 + B1/X). (**A**) GLUT1 d + 21 (R^2^ = 0.314, B0 = −30,557, B1 = 107,794), (**B**) GLUT3 d + 7 (R^2^ = 0.871, B0 = −495,507, B1 = 761,265), and (**C**) GLUT3/GLUT1 ratio d + 7 (R^2^ = 0.975, B0 = −0.432, B1 = 3.549). Reprinted from Eger et al. (2016), Copyright (2016) with permission from Elsevier.

Generally, dietary energy supply affects glucose oxidation and transport in leukocytes in ruminants [134,135] and provide hints regarding the special competition for this essential metabolite. Inversely, elevated plasma concentrations of the acute phase protein haptoglobin are associated with remarkable decreases in milk yield [81,136]. Anti-inflammatory treatments substantially increase lactational milk yield [137,138]. This demonstrates that inflammation has some kind of regulatory potential of on mammary glucose extraction. Still, the question ‘how nutrient partitioning is regulated when resources become scarce’ remains. In this regard, it was speculated that decreases in monocyte GLUT1 protein and mRNA expression after calving are due to lactogenesis [135]. In fact, Eger et al. demonstrated a direct negative correlation between lactose yield and overall expression of GLUT1 and GLUT3 as well as a decrease in GLUT3/GLUT1 ratio of monocytes with increasing lactose yield (Figure 3A–C) [96]. On the other hand, downregulation of some GLUT isoforms in the mammary gland was observed following LPS-induced mastitis during mid lactation [139]. However, mRNA abundance of mammary GLUT1 transporter, which is the most important one for lactose synthesis [140], does not decrease in cows submitted to a hyperinsulinemic–hypoglycemic clamp, not even when these cows were submitted to an additional intramammary LPS challenge [139]. As described above (Section 3.1), mammary extraction of glucose from the plasma pool is likely to not be limited by GLUT expression of these cells and the plasma concentration of glucose but rather depends on the rate of local blood flow resulting from the metabolic activity of the gland. In contrast to mammary epithelial cells, circulating immune cells rely on the rate of GLUT expression and increases in types of GLUT that are insulin-independent to cover their glucose demand.

Decreases in lactose yield as well as decreases in the mRNA-abundance of the ALA-subunit of lactose synthase were reported in hypoglycemic cows [139,141]. Similarly, reduced lactose content in milk following intramammary infection was reported [142,143] and could be a mechanism to save glucose for immune functions or/and to reduce substrate for bacterial growth during infection. On the other hand, Kreipe et al. showed that fat and protein percentages increased in hypoglycemic cows while energy-corrected milk did not differ significantly between hypoglycemic and control animals [141]. Thus, the extraction of glucose by the mammary gland might be unchanged during hypoglycemia, whereas glucose partitioning within the mammary gland is shifted from lactose synthesis towards glycolysis and pentose phosphate pathway to support protein and fat synthesis as was detected in bovine MEC exposed to various levels of glucose [144].

In fact, high-producing dairy cows were shown to be unable to reduce milk synthesis during early lactation in particular, while being able to reduce milk synthesis during induced energy deficiency at 100 days in milk, even if induced NEB was more severe compared to early-lactation NEB [132]. Accordingly, milk yield reductions following infusion of 100 µg LPS were found to be more pronounced in late lactation compared to early lactation [145,146]. Milk production of cows challenged with intramammary infusion of 30 cfu [147], 1 × 10^4^ cfu of diluted E. coli per quarter or 1000 µg LPS [148] decreased to low levels. In contrast, milk yield was unaffected by chronic and exponentially increasing intravenous infusions of LPS (0.017–0.148 μg/kg of body weight per hour from day 1 to 7) in a recently conducted study [149]. Daily subcutaneous injection of 3 µg/kg body weight of bovine tumor necrosis factor-α during the first week of lactation decreased milk yield only slightly (33.7 to 28.4 kg at highest dose) [149]. Osmotic TNFα pumps releasing 14 µg/kg body weight over 7 days implanted in adipose tissue in late lactation cows had no effect on milk yield [150].

Further evidence for an antagonistic, yet dysbalanced relationship between metabolic pathways involved in adaptation to lactation and adaptation to inflammation is derived from studies examining the effect of the characteristic endocrine alterations required for high milk yields. Compared to low or medium genetic merit cows, high genetic merit cows show lower plasma concentrations of glucose, insulin and IGF-1, as well as higher plasma concentrations of GH [151,152], while insulin resistance is increased [63,153,154,155]. As described above, hypoinsulinemia favors glucose uptake in both immune cells and MEC because these cells are not dependent on insulin whereas glucose uptake to insulin-dependent cells like adipose and muscle cells is reduced [67]. However, hypoinsulinemia also mitigates stimulating effects of insulin on the rate of glucose utilization and phagocytosis in immune cells [156,157]. Moreover, increased GH-resistance is associated to selection for milk production and might contribute to the dysbalanced allocation of resources between MEC and immune cells in dairy cows. While GH exerts its mammogenic and galactopoietic effects directly in the mammary gland, either through GHR or through mammary IGF-1 production [158,159], many of the immune-stimulating effects attributed to GH are mediated indirectly through induction of hepatic IGF-1 production [160]. However, IGF-1 production in the liver is blunted through hepatic GH-resistance during early lactation [47]. Interestingly, it was shown that different breeds selected for milk production (Holstein-Friesian and Guernsey) showed similar decreases in GHR1A mRNA expression [161], whereas a comparison between Holstein-Friesian and beef cattle revealed decreases in the expression of GHR1A in dairy cows only [162].

## 5. Management of High-Producing Dairy Cows that Risk Glucose Shortage

The management of high-producing dairy cows should aim for a maximal reduction in metabolic and environmental stress to reduce the energy demand of regulatory systems. Although the specific demand of an activated immune system is difficult to assess, tendencies may be estimated from plasma levels of inflammatory markers, as it has been recently suggested by Trevisi and Minuti [163]. Moreover, the amount of residual glucose left for life functions other than milk synthesis may be estimated by consideration of the glucose demand of quantifiable processes like milk synthesis on one hand and the amount of glucose supply from precursors (derived from feedstuffs and body tissue mobilization) and the hepatic gluconeogenic potential on the other.

Furthermore, dry off feeding and heifer management should be optimized, as it was repeatedly shown that nutrition during these life stages affects availability and allocation of nutrients during early lactation [164]. For instance, overfeeding cows by 50% of predicted requirements decreased postpartum plasma glucose and insulin while increasing glucagon, BHB, and NEFA concentrations after calving compared with cows fed a balanced energy diet during the dry period [165]. During lactation, feeding should be more adapted to meet the need of individual cows in their specific physiological and environmental condition. In particular, supply with glucogenic precursors should be optimized. Although Lucy et al. demonstrated the key role of glucose by showing that infusions of substantial daily doses of glucose (8500 to 1500 g/day) into early postpartum cows were able to completely reverse the hypercatabolic reaction (significant increases in blood concentrations of insulin and IGF-1 along with significant decreases in the concentrations of NEFA and BHB) [166], feeding glucogenic diets is unlikely to significantly reverse lactational energy partitioning, although controversial results can be found in the literature [167,168,169]. Certainly, nutritional interventions are limited through, e.g., careful use of grain in the diet, time to eat, rumen volume and liver function. There are reasonable doubts whether dairy feeding regimes can further optimize the supply with precursors and the potential of gluconeogenesis and thus increase total glucose availability. For the sake of animal health and welfare and the economic implications of production diseases, dairy farmers should consider a modest but precise reduction on the other side of the equation, i.e., apply management measures that decrease glucose output via milk during periods when dairy cows are challenged simultaneously by both high yields and infectious or non-infectious stressors. Possible management tools include a reduced milking frequency at the onset of lactation [170,171,172]. In fact, it has been demonstrated that reduced milking frequency reduces both milk yield and inflammation simultaneously [173]. Moreover, instead of implementing general strategies for a very heterogeneous target group, dairy cows should be assessed individually according to their status of NEB. Dairy cows with a high NEB should be allocated to a risk group and dealt with appropriately. In the long term, breeding should be redefined to include increased selection for persistence, lifetime performance and longevity while reducing emphasis on selection for milk yield and early-lactation performance in particular.

## 6. Conclusions

NEB is commonly thought to identify metabolically instable situations associated with increased risk of disease in dairy cows. However, overall energy balance disregards the reliance of immune cells on glucose as their essential metabolite and synthetic precursor. Although both proinflammatory signaling and lactational reprogramming promote several similar metabolic pathways with the aim of maximizing glucose availability to the respective cell types (lactocytes or leukocytes), adaptation to lactation clearly shifts nutrient partitioning to the favor of the mammary gland. Adaptations are mediated primarily by hormones of the somatotropic axis and affect both mobilization and allocation, including specific regulation of blood flow and receptor expression patterns in peripheral, mammary and immune tissues. Additionally, decades of performance-oriented selection of dairy cows enhanced these patterns substantially by increasing the amount of mammary epithelial cells as well as the metabolic and endocrine setup required to support the demand of these cells. Due to the central role of glucose for milk production and immune cell function, glucose balance is especially submitted to competitive allocation dynamics and is at risk of being overstressed in the early postpartum, high-producing dairy cow, as indicated by reduced responsiveness of lactose synthesis and milk yield to energy or glucose restriction, or other stressors. Therefore, we hypothesize that the uncoupling of the somatotropic axis in cows with high genetic merit for milk production implies, at least in part, an uncoupling of the mammary gland from life function trade-offs. To address possible impacts of glucose shortage on the immune defense, research should focus on the dynamics of glucose supply and demand of immune cells in high producing dairy cows during different periods of lactation.
